# A Case of Pseudo-Epilepsy of the Retina Caused by Hypermetropia

**Published:** 1887-01-01

**Authors:** F. C. Riley

**Affiliations:** New York


### A CASE OF PSEUDO-EPILEPSY OF THE RETINA CAUSED BY HYPERMETROPIA.

BY F. C. RILEY, M. D., OF NEW YORK.

Presumably, true epilepsy of the retina never occurs independent of the epileptic state, although an epileptoid condition of this membrane may recur and at regularly varying intervals, and with such demonstrative effect as to be very misleading if not sufficient to cause a complete error of judgment. Undoubtedly during all epileptic convulsions the vessels of the retina become emptied and blanched as soon as the cerebral circulation, as a whole, is interfered with, and remains so during the interval of unconsciousness. Were we to use the ophthalmoscope at such time we would find this condition existing, and the usual power entirely in abeyance. That an epileptoid manifestation can occur entirely independent of a true epilepsy and be dependent upon a cause remote from any of a neurotic nature, the history and subsequent developments of the accompanying case fully demonstrate. The peculiar symptoms here presented are of interest not only to the specialist in ophthalmic surgery, but to every physician, for to any one such cases may present themselves, and an appreciation of the danger attending such phenomena at the beginning will enable him to alleviate to a considerable degree by his prognosis, the anxiety of the sufferer.

Mrs. W., aged twenty-five, consulted me in the summer of 1885, recounting the following symptoms: Has always possessed good eyesight until recently. Was quite ill about eight months previous to parturition, which illness was prolonged by excitement consequent upon the illness and death of a member of the family. Supposed, however, to have fully recovered from the effects of the foregoing previous to the manifestation of any ocular symptoms.

During a period of two months prior to my examination, she had experienced more or less constant pain in the supra-orbital region, and at times all about the region of the eyes, especially when reading or sewing, the same generally subsiding soon after laying aside the book or needle. For the past two or three weeks there was experienced a sensation of fogginess before the eyes at intervals varying from a few hours to two days, both when regarding near or distant objects — these attacks increasing in severity until the right eye was incapable of distinguishing light from darkness; in fact, the patient became absolutely blind in the right eye, which condition lasted from two to ten or fifteen minutes and then passed off, generally through varying stages to a perfect restoration of sight.

The inadvertent closing of the left eye brought to her mind a sudden realization that the blindness was confined exclusively to the right eye, and subsequent repetitions demonstrated conclusively that the left eye was never thus affected. Fearing organic disease of the right which might lead to permanent blindness naturally caused much uneasiness and perturbation of mind.

An examination of the eye revealed the following:

Both eyes appear externally to be in normal condition. Insides dark; pupils regular and of same size; both respond perfectly and equally to light. O. D. V. = with X $\frac{1}{50}$ $\frac{XX}{XX}$; O. S. V. = with X $\frac{1}{50}$ $\frac{XX}{XX}$, showing a manifest hypermetropia of $\frac{1}{50}$. Slight insufficiency of internal rectus of right eye noticed when attention was concentrated upon a near object. Not having a set of prisms at hand, the degree of adduction and abduction was not noted. With the ophthalmoscope the left eye was ascertained to be in a perfectly normal state. In the right eye was noticed slight engorgement of the veins, they being somewhat distended and tortuous, especially near the margin of the disc. Otherwise the fundus presented a perfectly normal appearance.

The patient had, therefore, all the symptoms of quinine epilepsy of the retina, with no other history of the disease and no abnormality to mention except the far-sightedness.

To prevent the return of the epileptical manifestations, she was directed to abstain from all work necessitating the use of the eyes in a condition of active accommodation, and to use a pair of glasses (X 1-60), representing almost the whole amount of her manifest hypermetropia.

The following day an attack occurred, which lasted but a moment, and did not produce blindness.

The second day a sense of fogginess of short duration was noticed, but no special inconvenience followed.

On the third day the glasses (X 1-60) were received and immediately adopted, since when no recurrence of the epileptoid attacks have supervened, and with their use, in conjunction with a short course of faradization of the internal rectus muscle complete relief from all pain, inconvenience and interference of vision has ensued. The lady recently informed me that she had not since experienced any untoward symptoms, and that with the aid of the glasses she is enabled to work steadily for hours at the finest kinds of needle-work, without experiencing the slightest sense of fatigue about the eyes.

The history of such cases demonstrate most conclusively the necessity of the use of correcting lenses in order to prevent the possibility of overtaxation of the accommodative power which generally occurs in the hypermetropic state unless glasses are worn.

This also brings to notice the diversity of manifestations that may supervene upon but a slight degree of refractive error.—*Med. and Surg. Rep.*

